# Recent advances in canola meal utilization in swine nutrition

**DOI:** 10.1186/s40781-016-0085-5

**Published:** 2016-02-16

**Authors:** G. Mejicanos, N. Sanjayan, I. H. Kim, C. M. Nyachoti

**Affiliations:** Department of Animal Science, University of Manitoba, Winnipeg, MB R3T 2 N2 Canada; Department of Animal Resource & Science, Dankook University, Cheonan, Choognam South Korea

**Keywords:** Canola meal, Nutritive value, Pigs

## Abstract

Canola meal is derived from the crushing of canola seed for oil extraction. Although it has been used in swine diets for a long time, its inclusion levels have been limited due to concerns regarding its nutritive value primarily arising from results of early studies showing negative effects of dietary canola meal inclusion in swine diets. Such effects were attributable to the presence of anti-nutritional factors (ANF; notably glucosinolates) in canola meal. However, due to advances in genetic improvements of canola that have led to production of cultivars with significantly lower ANF content and improved processing procedures, canola meal with a superior nutritive value for non-ruminant animals is now available. Therefore, the aim of this paper is to review the recent studies in the use of canola meal as feedstuff for swine, the factors influencing its use and the strategies to overcome them. First a historical overview of the development of canola is provided.

## Background

Canola is an offspring of rapeseed which belongs to the cabbage family or Brassicas. The genus *Brassica* also contains plants such as cabbage, radish, kale, mustard and cauliflower [10]. Rapeseed oil contains around 25-45 % erucic acid whereas the meal contains about 110-150 μmoles/g of aliphatic glucosinolates [12]. Rapeseed was cultivated more than 3000 years ago in India and 2000 years ago in China and Japan. The development of steam power resulted in better industrial acceptance of rapeseed. It was introduced to Canada between 1936 and early 1940s as a method of diversifying crop production, especially for the Prairie Provinces [10, 30, 69]. The fuel shortage caused by World War II led to the increased production of rapeseed. However, with the switch to diesel engines, and also the ban of the use of rapeseed for human consumption by the USA in 1956, the demand for rapeseed declined [95].

**Fig. 1:**
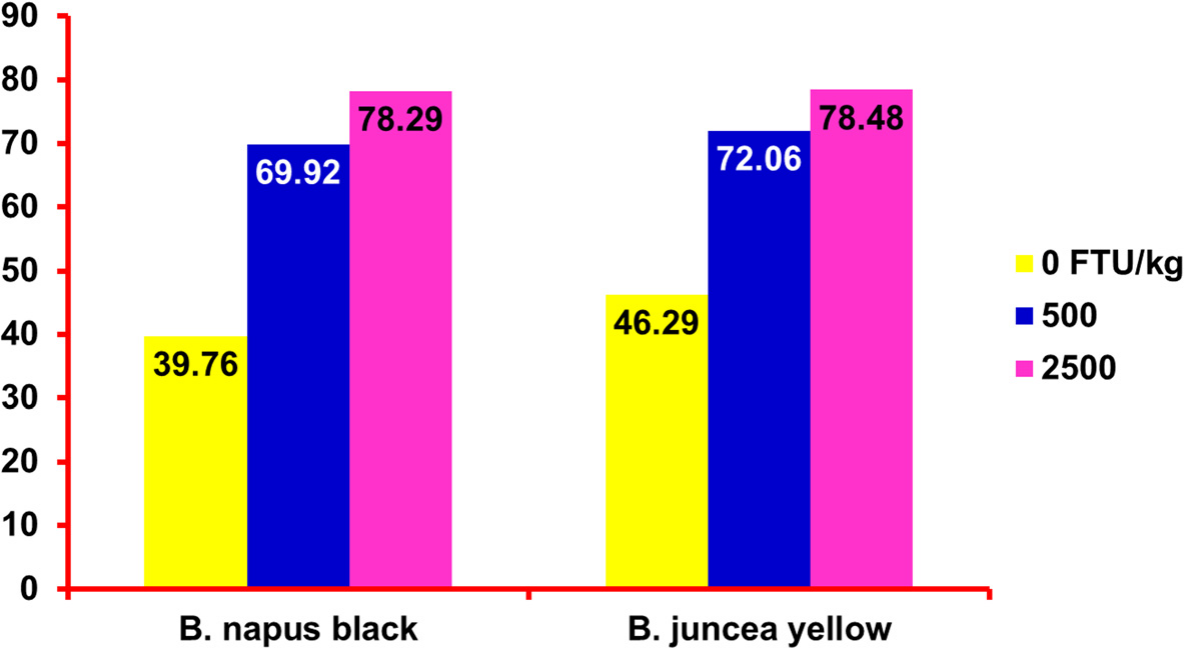
Effect of phytase supplementation on standardized total tract of digestibility of phosphorus in two types of canola meal fed to growing pigs (Adapted from [3])

Rapeseed contains high levels of glucosinolates, which can be hydrolyzed by the enzyme myrosinase to release products with goitrogenic effects that interfere with iodine metabolism and therefore affect the functioning of the thyroid gland and consequently animal performance [53]. To address these effects, plant breeders worked to develop rapeseed cultivars with low glucosinolate content in the meal and low erucic acid content in rapeseed oil. The first low-erucic acid rapeseed was developed in Canada by Dr. Baldour R. Stefansson of the University of Manitoba, who has been referred to as “The father of canola” because of his contribution to the development of low-erucic acid type rapeseed. In early 1960s, he surveyed over 4000 lines of rapeseed from all over the world and identified low-erucic acid lines which were then used in the breeding programs at the University of Manitoba and also by Dr. Keith Downey at the Agriculture Canada Research Station in Saskatoon. In 1968, the first low-erucic acid cultivars Tanka, Target and Turret were released and produced in Canada [10, 85]. By 1974, Dr. Stefansson released the first double zero rapeseed cv. Tower [10].

In 1979, all double low cultivars produced in Canada were named as Canola [10]; the name of canola is a contraction of Canada and “ola” that refers to “oil low acid” [22]. The name was used to differentiate canola from the high-glucosinolate, high-erucic acid rapeseed. The name canola refers to “Seeds of the genus *Brassica* (*Brassica napus*, *Brassica rapa* or *Brassica juncea*) from which the oil shall contain less than 2 % erucic acid in its fatty acid profile and the solid component shall contain less than 30 micromoles of any one or any mixture of 3-butenyl glucosinolate, 4-pentenyl glucosinolate, 2-hydroxy-3 butenyl glucosinolate, and 2-hydroxy-4-pentenyl glucosinolate per gram of air-dry, oil-free solid” [22]. In the international community canola is also known as “double zero”, “Zero-zero” or “double low” rapeseed. Canola is currently the leading oil seed crop in Canada with an annual production of over 15 million tonnes [23] and the importance of its meal as a protein supplement is second only to soybean meal. During crushing, canola seed yield 42 % of oil, which is used as vegetable oil for human consumption and 58 % meal, which is used as a protein source in animal feed [94]. The aim of this article is to review the recent studies in the use of canola meal as feedstuff for swine, the factors influencing its use and the strategies to overcome them.

### Chemical and nutritive value of canola meal

Earlier studies with different types of canola demonstrated that black and yellow seeds differ significantly in their chemical and nutritive composition, particularly in the contents of oil, crude protein (CP) and fiber [56, 82]. As can be seen from Table 1, CP content of three different types of canola meal (CM) differed significantly, with *B. juncea* showing the highest protein content of 42.3 %, followed by 41.0 % in yellow seeded *B. napus* and 36.9 % in *B. napus* black (as is basis). Furthermore, distinctive differences can be observed between cultivars in terms of NDF, ADF, NSPs, lignin and pholyphenols, phosphorus, etc. The oil extraction process of the seeds would also affect the CP content with oil-expelled CM containing 35.2 %, while pre-press solvent extracted meal containing 37.5 % (as-fed basis) [65]. Other factors that affect the protein content of CM are the environmental conditions during the growing season. Tipples [92] found that over the 10 years, from 1978 to 1987, the CP content of CM ranged from 36 to 41 %. Bell et al. [14] found that location is another factor that can affect mineral content of *B. napus*, *B. rapa *and *B. juncea*.

**Table 1:** Chemical composition of meals derived from black- or yellow-seeded *B. napus* canola and canola quality *B. juncea* (% as is basis) ^a^

Component	*B. napus* “black”	*B. napus* “yellow”	*B. juncea* “yellow”
Crude protein	36.9	41.0	42.3
Fat	3.8	3.7	3.4
Ash	7.1	7.9	6.6
Sucrose	6.3	8.4	7.6
Dietary fiber fractions			
Acid detergent fiber	17.0	12.0	9.7
Neutral detergent fiber	23.6	16.4	15.9
Non-starch polysaccharides	17.0	21.1	19.4
Total fiber %	30.1	27.1	25.5
Lignin and polyphenols	10.3	2.7	4.0
Glycoprotein	2.8	3.2	2.1
Phosphorus (P)	0.95	1.25	1.04
Phytate P	0.56	0.80	0.58
Non-phytate P	0.39	0.44	0.46
Calcium	0.67	0.55	0.76
Glucosinolates, μmol/g ^b^	9.2	13.5	12.2

^a^Adapted from Mejicanos [56]; ^b^Includes gluconapin, glucobrassicanapin, progoitrin, gluconapoleiferin, gluconasturtin, glucobrassicin, and 4-hydroxyglucobrassicin

#### Protein and amino acid source

It has been documented that the meal from yellow seeded *B. juncea* and *B. napus* yellow contains more CP (DM basis) in comparison with the conventional *B. napus* black; 43.4 and 47.2 vs. 41.1 % [93]. CM contains a well-balanced amino acid (AA) profile and when compared to soybean meal (SBM), it contains less lysine, but more sulphur AA (i.e. methionine and cysteine) ([67]a). CM contains approximately 2 % methionine as a percent of total protein, while SBM has 1.5 %. However, CM has lower amount of lysine compared to SBM. It also contains 10 % lower available lysine compared to SBM [76]. Therefore, both meals complement each other when used in rations for livestock and poultry [40]. It has been reported that there is a negative relationship between protein and dietary fiber content of meals derived from black and yellow seeded *B. napus* canola [82], such differences will affect the percentage of AA content of the different cultivars. Removing fiber from the meal would translate into fractions with higher CP and AA content. For example, Mejicanos et al. [57]. evaluated the nutritive value of dehulled CM and observed that with the reduction of fiber, the CP and AA contents were increased. Table 2 shows the AA composition of meals derived from black and yellow *B. napus,* and yellow *B. juncea* and the corresponding dehulled fraction 1 produced by sieving; e.g. lysine increased from 2.02 to 2.26 %, from 1.91 to 2.34 and from 1.95 to 2.29 % for *B. napus* black, *B. napus* yellow and *B. juncea* meal respectively. Methionine increased from 0.68 to 0.81, 0.63 to 0.71 and 0.66 to 0.83 % for *B. napus* black, yellow and *B. juncea*, respectively. Conditions in the processing plants also affected the quality of CM, and in that regard Adewole et al. [2] reported significant variations in AA content (P < 0.05) of CM from different processing plants across Canada; e.g. arginine, lysine, methionine, and threonine averaged 2.22, 1.78, 0.52, and 1.07 %, respectively, and ranged from 2.00 to 2.44 % for arginine, 1.61 to 1.96 % for lysine, 0.45 to 0.63 % for methionine, and 0.94 to 1.34 % for threonine. The study also reported that pelleting significantly reduced the AA content of the meal. Results reported by Adewole et al. [2] indicates that standardized Ileal digestibility (SID) of arginine, lysine, methionine and threonine averaged 87.5, 78.8, 85.4 and 74.8 %, respectively. Table 3 shows SID values of solvent extracted CM (SECM) and expeller extracted CM (EECM) fed to growing pigs as reported by Woyengo et al. [96], Maison and Stein [50], Seneviratne et al. [79] and Sanjayan et al. [75].

**Table 2:** Amino acid composition of conventional *B. napus* “black” canola meal, *B. napus* yellow meal and canola-type *B. juncea* yellow mustard meal, and their corresponding dehulled fraction 1 produced by sieving (%, as-is basis)^a^

	*B. napus* “black”		*B. napus* “yellow”		*B. juncea* “yellow”	
Amino acid	Parent meal	Dehulled fraction 1	Parent meal	Dehulled fraction 1	Parent meal	Dehulled fraction 1
Alanine	1.49	1.76	1.56	1.89	1.72	2.05
Arginine	2.28	2.77	2.08	2.63	2.85	3.60
Aspartate	2.62	3.01	2.30	2.89	3.34	3.87
Cysteine	0.80	0.92	0.91	0.94	0.70	0.85
Glutamine	6.60	7.81	5.91	7.44	7.26	8.49
Glycine	1.85	2.19	1.45	1.85	2.16	2.56
Histidine	1.18	1.37	1.10	1.35	1.31	1.51
Isoleucine	1.21	1.46	1.06	1.34	1.21	1.81
Leucine	2.43	2.92	2.31	2.86	2.76	3.52
Lysine	2.02	2.26	1.91	2.34	1.95	2.29
Methionine	0.68	0.81	0.63	0.71	0.66	0.83
Phenylalanine	1.40	1.69	1.31	1.61	1.53	1.98
Proline	2.54	2.89	2.44	2.85	2.77	2.93
Serine	1.69	1.93	1.63	1.99	1.94	2.18
Threonine	1.62	1.85	1.33	1.66	1.82	2.14
Tyrosine	0.93	1.11	0.84	1.06	1.05	1.34
Valine	1.66	1.95	1.54	1.90	1.62	2.35

^a^Adapted from Mejicanos [56]

**Table 3:** Standardized ileal digestibility (%) of amino acids in canola meal fed to growing pigs

Item	Expeller extracted			Solvent extracted		
	[96]	[79]	[50]	[96]	[75]	[50]
Essential						
Histidine	84.7	81.7	83.8	78.1	87.1	82.0
Isoleucine	85.4	74.3	77.7	78.1	79.7	75.9
Leucine	87.2	78.8	81.6	79	80.3	79.3
Lysine	70.7	73.2	74.7	66.6	78.9	70.6
Methionine	87.4	83.9	87.1	84.1	84.2	84.5
Phenylalanine	90.4	78.0	81.1	90.4	70.8	78.2
Threonine	79.5	67.6	74.0	72.1	77.1	73.0
Tryptophan		83.9	83.4			82.6
Valine	83.8	70.5	75.9	76.7	78.5	74.4
Conditionally Essential						
Arginine	91.7	83.1	89.4	86.2	90.3	86.3
Cysteine	80.1	72.7	72.9	79.3	79.8	73.2
Tyrosine	98.2	75.1	75.6	93.3	78.7	74.7
Non-essential						
Alanine	85.1	72.1	80.2	76.3	78.2	75.8
Aspartate	82.2	72.0	77.8	75	77.8	71.8
Glutamate	91.6	84.3	85.9	86.9	88.3	83.4
Glycine	86.2	63.6	78.6	82.2	76.5	78.1
Serine	76.7	70.6	76.7	76.7	80.7	75.7

Woyengo et al., [96]; Seneviratne et al., [79]; Maison and Stein [50]; Sanjayan et al., [75]

#### Energy source

One of the main factors that limit the nutritive value of CM is its low digestibility of energy which is a reflection of its high crude fibre content [88]. Compared to soybean, canola contains a higher amount of oil with many cultivars containing between 40 and 45 % oil on a dry matter basis [32]. The energy content of CM can differ between samples obtained from different crushing plants due to the oil extraction process, i. e. expelled CM contains residual oil at average levels of 9.7 %, compared to 3.2 % for the pre-press solvent extracted meal [65]. The oil content of the meal from the pre-press solvent extraction process would also be affected by the amount of gums added back to the meal following oil refining. As indicated by Bell [12], gums may contain about 50 % of canola oil and such oil is expected to increase the ME values of the meal.

Theodoridou and Yu [91] evaluated the effect of processing conditions on the nutritive value of canola meal and reported significant differences between CM from black- and yellow-seeded *B. napus* for the basic nutrients, except ash. The differences between yellow and black canola included NDF, ADF, CP, and condensed tannins. Yellow-seeded CM showed higher values for CP, total digestible CP, and lower fiber content [12, 82]. The differences between CM from different cultivars of canola are illustrated in Table 1. Sucrose content for yellow seeded *B. napus* was higher, and averaged 8.4 %, while the mean values for *B. juncea* and *B. napus* black were 6.3 and 7.6 %, respectively. In the case of non-starch polysaccharides, yellow-seeded *B. napus* reported higher values and averaged 21.1 %, whereas values for *B. juncea* and *B. napus* black averaged 19.4 and 17.0 %, respectively. Total dietary fiber was lower in *B. juncea* CM, and averaged 25.5 %; 27.1 % for yellow-seeded *B. napus* whereas *B. napus* black had 30.1 %. In the case of expelled meal which contains an average 10.0 % of ether extract, the values reported for GE, DE, ME and NE averaged 4873, 3779, 3540 and 2351 kcal/kg, respectively. For pre-press solvent extracted CM, which contains less ether extract (3.2 % on average), the values average 4332, 3273, 3013 and 1890 kcal/kg, respectively [65]; whereas the values for yellow seeded *B. napus* averaged 3.965, 3248, 3009, and 2102 kcal/kg, respectively; the values for yellow *B. juncea* averaged 4037, 3392, 3224 and 2340 kcal/kg, respectively [38].

Dehulling of canola can result in a higher energy meal, as shown by research on tail end dehulling of pre-press solvent extracted CM from black and yellow seeded *B. napus* and canola quality *B. juncea;* dehulling resulted in low fiber high protein fractions Fine 1 and Fine 2. Compared to their parent meals, the content of total dietary fiber in the fractions decreased from 30.1 to 21.4 and 26.7 % for conventional CM, from 25.5 to 15.3 and 18.7 for yellow-seeded CM, and from 27.1 to 21.6 and 23.4 for *B. juncea* meal, respectively [56]. The complete removal of the hulls of canola would result in high protein-high energy meal with 47.8 % protein, 10.8 % NDF, 6.6 ADF. [25].

#### Vitamins and minerals source

Canola meal is a rich source of most of the minerals [12]. Compared to soybean meal, CM has relatively high amounts of Ca, P, S, Mg, Mn and Se, but K and Cu contents are lower Table 4 shows the chemical composition of CM compared to soybean meal [12, 40, 80]; such values are in accordance with National Research Council. Nutrient requirements of swine. 11th Rev. Ed et al. [65], However, the presence of phytic acid and high fibre in the meal reduces the availability of most of the minerals. Although the availability of most of the minerals is low in CM, it has high amounts of available Ca, Mg and P compared to soybean meal as shown in Table 4. Canola meal contains considerably high amount of phytate-bound phosphorus in proportion to total phosphorus and which ranges from 36 % to over 70 % [40]. Due to this reason bioavailability of phosphorus has been estimated to be around 30 to 50 % of the total phosphorus in CM [35]. Compared to SBM, CM is a richer source of vitamins such as biotin, niacin, choline, thiamin, Vitamin B6 and niacin. However, pantothenic acid content is lower in CM [28, 65].

**Table 4:** Chemical composition of canola meal compared to soybean meal

Components	Canola meal	Soybean meal
Dry matter, %	90.0	90.0
Crude protein, %	36.5	45.6
Ether extract, %	3.6	1.3
Gross energy, MJ/kg	18.6	20.1
Carbohydrates, %		
Starch	2.5	0.7
Sucrose	6.0	6.2
Sugar	7.7	6.9
Oligosaccharide	2.5	5.3
Fibre, %		
Crude fibre	11.6	5.4
Non-starch polysaccharide	18.0	17.8
Neutral detergent fibre	26.0	12.0
Acid detergent fibre	18.2	7.5
Total dietary fibre	31.7	21.8
Amino acids, %		
Arginine	2.04	3.23
Lysine	2.00	2.86
Threonine	1.57	1.74
Methionine	0.74	0.65
Cysteine	0.85	0.67
Tryptophan	0.48	0.64
Minerals, %		
Calcium	0.7	0.3
Phosphorus	1.2	0.7
Magnesium	0.6	0.3
Sodium	0.08	0.01
Potassium	1.29	2.0
Vitamins, mg/kg		
Biotin	1.0	0.3
Folic acid	2.3	1.3
Niacin	169.5	29.0
Pantothenic acid	9.5	16.0
Riboflavin	3.7	2.9
Thiamine	5.2	4.5

Bell [12], Simbaya [80], Khajali and Slominski [40]

### Factors affecting feeding and nutritive value of canola meal for swine

There are several factors that limit the use of CM, especially in monogastric animal nutrition. When compared with SBM, CM contains higher contents of dietary fiber, glucosinolates, sinapine, phytic acid, phenolic components such as tannins, lower metabolizable energy, with less consistent AA digestibility and less than optimum electrolyte balance due to high sulfur and low potassium contents [40]. Among these, fibre, glucosinolates, phytic acid and and sinapine are considered to be the main antinutritional factors in CM.

#### Fibre

Fiber content in CM is 3 times higher than SBM [12], which is the result of a large proportion of hulls relative to seed size. The hull represents 16.8 % to 21.2 % of the seed mass [25], but increases to about 30 % of the meal weight after oil extraction, which is the main reservoir for non-starch polysaccharides (NSP) and lignin. Low levels of DE and ME in CM is due to the high level of fiber [12]. High protein soy and 44 % soy with hulls added back contain around 4 % and 7.5 % fibre, respectively, whereas CM has more than 10 % crude fibre [32]. CM contains cellulose (4-6 %), non-cellulosic polysaccharide (13-16 %), lignin and polyphenols (5-8 %) and proteins and minerals associated with the fibre fraction as the major fibre components [81]. Previous studies demonstrated that yellow-seeded meal has low amount of fibre compared to black-seeded meal. For instance, ADF and NDF contents of *B. juncea* (9.7 % and 15.9 %) are lower compared to those (17.0 % and 23.6 %) of *B. napus* black as shown in Table 1 [56].

Fibre mainly contains NSP, lignin associated with polyphenols, polyphenol glycoproteins and minerals associated with fibre [80]. Non-starch polysaccharide components of CM are shown in Table 5. Pectic polysaccharidies are present in CM as a non-cellulosic polysaccharide, which is indicated by the presence of uronic acid [81]. Arabinose, xylose, galactose and rhamnose are the main components of galacturonic acid. Part of the arabinose and galactose were derived from arabinan and/or arabinogalactan. Presence of xylose indicates the presence of xylan and xyloglucans. Xyloglucans contain xylose, glucose, galactose and fucose [81]. Cellulose, arabinose, arabinogalactan and pectins are the major NSP components in CM [41, 59, 81]. In the study by Meng and Slominski [58] it was reported that CM contained 174.5 mg/g total NSP of which 14.3 mg/g was water soluble.

**Table 5:** Non-starch polysaccharides components of canola meal (mg/g)

Component	Black *B. napus*	Yellow *B. juncea*	Yellow *B. napus*
Rhamnose	1.2	1.2	1.0
Fucose	1.0	0.8	0.8
Arabinose	22.9	24.1	24.8
Xylose	9.1	7.5	10.3
Mannose	2.6	1.5	2.1
Galactose	7.9	7.7	8.8
Glucose	29.6	27.6	27.2
Uronic acids	26.6	30.4	26.5

Adapted from [82]

#### Glucosinolates

Glucosinolates (GLS) are sulphur-containing secondary plant metabolites found mainly in the order Capparales known also as Brassicales, which contain plants of the family *Brassicaceae* that includes the genus *Brassica* (rapeseed, mustard, and cabbage) [27, 40]. Intact GLS do not cause any harmful effects to animals, however, the break down products of GLS either by enzyme myrosinase or by non-enzymatic factors such as heat, low pH, anatomical and physiological structure of the gastrointestinal tract, digesta transit time and microbial activity cause harmful effects to animals [12]. Depending on the nature of GLS, reaction condition and concentration, the break down products- thiocyanate, isothiocyanate, oxazolidinethione (goitrin) and nitriles may be formed and impair not only feed intake (due to their bitter taste) and growth performance but also affect thyroid function by inhibiting thyroid hormone production and impair liver and kidney function [12, 20, 61]. Previous studies show that growing pigs can tolerate a maximum of 2.0-2.5 μmol/g of glucosinolates in the diet [12, 74, 77].

Glucosinolates are considered an anti-nutritional factor present in CM. Rapeseed meal contained 110-150 μmol/g of GLS [12]. However, through plant breeding techniques new canola varieties have been developed with low level of GLS (<30 μmol/g). In a survey from crushing plants across Canada, the level of GLS in CM was reported to average 3.9 μmol/g [73]. Reports from France show that the level of GLS in double-zero rapeseed averaged 10 μmol/g [44]. Mejicanos [56] reported GLS values of 9.2 and 12.2 for *B. napus* black and *B. juncea*, respectively. CM contains two types of GLS, aliphatic (85 %) and indolyl (15 %) [67]. Gluconapin, glucobrassicanapin, progoitrin and napoleiferin are the major aliphatic GLS present in CM of which progoitrin is the major factor which is responsible for the anti-nutritional effect [37, 80]. Table 6 shows the GLS content of *B. napus* black and *B. juncea* meals and its dehulled Fractions 1 and 2. As can be observed, dehulling did not increase significantly the content of GLS; however, the content of gluconapin was higher in *B. juncea* meal (10.1 μmol/g) compared to *B. napus* meal (2.1 μmol/g) which can affect palatability especially in weaned pigs. Landero et al. [47] found that the level of GLS in *B. juncea* of 10.8 μmol/g decreased ADG as the levels of inclusion of CM in the diet increased, which indicates that piglets are very sensitive to GLS present in *B. juncea* meal. The reduced growth performance of weaned pigs could be the result of high sensitivity of young pigs to GLS of *B. juncea* meal, especially gluconapin which is the most abundant and responsible for growth depression in weaned pigs. Mejicanos [56] found decreased feed efficiency in weaned pigs fed pre-starter diets containing dehulled CM from *B. Juncea,* which can be attributed to increased amounts of the glucosinolate gluconapin. In the same experiment, Mejicanos et al. [56] reported that when pigs were fed diets containing CM from *B. napus* black, feed efficiency increased compared to pigs fed diets containing *B. juncea* CM or diets containing the control SBM.

**Table 6:** Glucosinolates content of *B. napus* black and *B. juncea* yellow meals and their respective dehulled Fractions 1 and 2 (μmol/g, as-is basis)

Glucosinolate	*B. napus* “black”			*B. juncea* “yellow”		
	Parent	Dehulled fractions		Parent	Dehulled fractions	
	Meal	1	2	Meal	1	2
Gluconapin	2.1	2.6	2.3	10.1	11.2	11.2
Glucobrassicanapin	0.3	0.3	0.3	0.8	0.9	1.0
Progoitrin	5.1	5.7	5.3	0.8	0.9	1.0
Gluconapoleiferin	0.2	-	0.3	-	-	-
Glucobrassicin	0.4	0.3	0.4	0.1	0.1	0.1
4-Hydroxyglucobrassicin	1.2	0.8	1.1	0.3	0.3	0.4
Total glucosinolates	9.2	9.6	9.6	12.2	13.5	13.6

Source: [56]

#### Phytic acid

Phytic acid [myo-inositol (1, 2, 3, 4, 5, 6-hexakis dihydrogen phosphate)] is the storage form of phosphorus in grains and oil seeds. Although its role in animal nutrition is not completely understood, it is considered an anti-nutritional factor [40]. It is present in CM at levels of 4-6 % and reduces its nutritional value by binding to multivalent cations like Zn, Ca, and Fe and thus reduces their bioavailability [5]. Woyengo and Nyachoti [97] concluded that phytic acid can affect animal performance by reducing nutrient digestibility through binding to nutrients, the digestive enzymes or both which, in turn, would result in increased endogenous loses of amino acids. A standard diet may contain 10 g/kg of phytic acid (2.8 g of phytate P/kg) and as much as 60 % of this may be hydrolyzed by microbial phytase, and absorbed by the terminal ileum. [1]. A recent study with CM from *B. napus* black and *B. juncea* by Adhikari et al. [3]*,* reported true total tract digestibility of phosphorus (TTTD) values of 33.3 and 32.0 % respectively, while standardized total tract digestibility (STTD) values were reported to be 31.0 and 28.3 %; the study reported endogenous loses of phosphorus averaging 665 ± 0.03 mg/kg DMI. Another study by Liu et al. [48] found similar results comparing two diet types in the estimation of true digestibility using the regression method and reported values to be 30.19 and 27.22 % for pigs fed a semi-purified diet and practical diet, respectively.

#### Tannins

Tannins in canola are found mainly in the hulls and dark-colored hulls contain more tannins than yellow hulls [33, 87, 98]. Insoluble tannins (i.e., proanthocyanidins) are predominant in canola and responsible for the dark color of the seeds. It has been demonstrated that adding soluble tannins to broiler diets resulted in growth depression [52]. However, tannins present in canola are basically water-insoluble and are located in the hulls and thus may have minimal effect on the nutritive value of canola [40]. Environmental growing conditions can affect the content of tannins [63]. Research on the effect of tannins on growth performance and intestinal ecosystem in weaned pigs has demonstrated some improvement in feed efficiency, which indicates that tannins may have beneficial effects, not just anti-nutritional effects [16]. Khajali and Slominski [40] reported that tannins have the potential to bind with protein and proteolytic enzymes in gastrointestinal tract, thereby reducing the protein digestibility.

#### Sinapine

Sinapine is the choline ester of sinapic acid [19], which is the most abundant phenolic ester in rapeseed; sinapine is a bitter tasting phenolic compound which is widely distributed among plants of the Cruciferae family, and therefore it would contribute to the unpleasant and bitter flavour of glucosinolate-free rapeseed products and its presence may limit feed intake [63]. Brand et al. [17] reported differences in the sinapine content of different canola cultivars, with a mean value of 9.95 mg sinapine/g grain and values ranging from 7.72 to 11.53 mg sinapine/ g grain. Research in Germany is underway to reduce the levels of sinapine in rapeseed/canola by developing low-sinapine varieties with yellow-seeded and low-fiber characteristics [68]. Sinapine levels have been reduced by up to 71 % and seeds with content of 2.4 mg/g as compared to control with 7.5 mg/g have been developed [36].

### Means of improving nutritive value of canola meal for swine

#### Meal production procedure

Canola meal, a co-product of canola oil crushing industry, is produced when oil is extracted using any one of the three main procedures. These includes, solvent extraction (where oil is extracted from the meal by physical expeller extraction followed by solvent washing), expeller pressed (where oil is physically extracted using heat) and cold pressed (where oil is physically extracted without heat treatment) [49].

The solvent extraction method, which is the most common and efficient method of oil extraction results in a meal that has less than 5 % residual oil [83]. The solvent extracted meal is placed into the desolventizer-toaster in which the solvent is removed by the use of steam which provides heat to vaporize the hexane. During this process the meal is heated to 95-115 ˚C and moisture content increased to 12-18 %. The desolventized meal is then toasted on heated metal plates. The final products contain 10 % moisture and less than 1 % oil content [49]. In the processing plant some of the canola oil refining products including gums and soap stocks may be added into the meal to increase the energy value and meal quality. Canola oil also can be extracted using expeller pressed method where the oil and meal is physically extracted with added moisture of less than 12 % and heat of up to 160 ˚C, but this method is less efficient and result in a meal with higher residual oil content (8-15 %) [21, 83].

The processing method used to extract canola oil would affect the quality of the meal, and in the case of solvent extraction, Newkirk et al. (2002) demonstrated that prior to desolventizing/toasting, processing has no effect on apparent ileal digestibility of AA, except for cysteine and serine. However, it was found that meal desolventization/toasting significantly decreases protein and amino acids digestibilities, especially lysine. Such detrimental effects are caused by Maillard reactions which would lead to the formation of aldose products of AA which are not effectively utilized. Adewole, et al. [2] also indicated that the nutritive value of CM, particularly, digestibility can be enhanced or diminished by processing conditions, as excessive heating during pre-press solvent extraction may result in reduced digestibility of AA, particularly lysine. Also, it was indicated that dietary fiber and corresponding low glucosinolate content observed in some crushing plants could have been caused by CM overheating. Schöne et al. (2015) evaluated toasting and AA availability of rapeseed meal in pigs and concluded that the improved acceptance of longer heated meal with lower GLS content is compromised by decreased content of limiting AA such as lysine and also by lower SID of most AA.

#### Dehulling procedure

According to studies conducted in INRA, France more than 70 % of rapeseed fiber is present in the hulls; consequently, the removal of the hulls would improve the quality of the meal (Carre, 2009). Several seed dehulling processes have been developed. Reichert et al. [72] developed a tangential abrasive dehuller device (TADD) consisting of an abrasive disk rotating horizontally, and a stationary lid with several grain cups over the rotating disk. The abrasive disk set to 80 degrees was found to be optimal for canola dehulling. Such a process, however, may require pre-conditioning of the seed to maximize the percentage of hull removal [90]. The French Institute for Oilseeds owns a patent for a dehuller that works based on a centrifugal propeller to separate the embryo and the hull fractions [86]. Dehulling can be done using an abrasive dehuller which requires conditioning of the canola seeds, and the dehulling index is variable depending on the time of moistening and heating, which makes the commercial application unpractical [39]. Other methods for dehulling (i.e., rolling) have been described but have not been shown to be very efficient.

Clark et al. [29] assessed tail-end dehulling of CM in broilers, the method used involved the addition of moisture up to 16 %, milling using a disc mill with 0.008” gap, and sieving trough a 70 mesh screen (250 μm) in order to obtain 2 fractions, one being partially dehulled CM with high protein and reduced fiber contents and the other, a coarse fraction, with partly elevated fibre and protein contents. Dehulling increased the protein and AA contents of dehulled meals, CP and lysine increased in the range from 0.4 to 10.9 % and 1.2 to 17.5 %, respectively with an average of 5 % for both, whereas the increase in crude fat was 2.1 to 56 %, and averaged 23 %. Kracht et al. [43] observed that following dehulling the CP content of CM increased from 39.6 to 42.4 % (DM basis). It was also found that the amounts of AA per kg of meal increased following dehulling by 11 %, with lysine increasing by about 5 % and methionine and cysteine by 26 %. Mejicanos et al. [55] evaluated tail end dehulling using pre-press solvent extracted meal*,* and obtained 2 dehulled fractions, Fine 1 and Fine 2; when the fractions were compared to the corresponding parent meal it was observed that the values of crude protein had increased from 36.8 to 42.0 and 39.6 % for the conventional *B. napus* black meal; from 41.0 to 43.6 and 43.0 % for yellow-seeded *B. napus* meal; and from 42.3 to 47.9 and 46.8 % for *B. juncea* meal (as-is-basis). Table 2 shows that the AA contents of the dehulled fraction 1 were higher than those in the corresponding parent meals. Methionine increased from 0.68 to 0.81 % for *B. napus* black, from 0.63 to 0.71 % for *B. napus* yellow, and from 0.66 to 0.83 % for *B. juncea*. Lysine also increased from 2.02 to 2.26 % for *B. napus* black, from 1.91 to 2.34 % for *B. napus* yellow, and from 1.95 to 2.29 % for *B. juncea*. Mejicanos [56] also indicated that GSL content was not significantly increased in the dehulled fractions, but in the case of *B. juncea* meal a different GSL profile was observed; gluconapin was reported as being 10.1 μmol/g (as-is basis) whereas *B. napus* black had 2.1 μmol/g (as-is basis). Table 6 shows the GSL content of *B. napus* black and *B. juncea* meals and their respective dehulled fractions 1 and 2.

Vibro-separation for meal classification has also been used in Alberta, Canada. Reducing the particle size by grinding of solvent-extracted *B. juncea* meal was effective in reducing the NDF content from 22.7 % for fractions over 850 microns to 11.8 % for fractions under 425 microns [15]. Another method of tail-end dehulling is “Air classification” which utilizes the difference in particle size/density (kg/m^3^) between hulls and embryo [90]. The hulls of canola are rich in fiber which is denser than the oil free cotyledons, so these seed components partially fractionate in a stream of air allowing air classification to separate CM into low-fiber, light-particle fraction and a high fiber, heavy-particle fraction which can be of interest for the feeding monogastric and ruminant species, respectively. Air classification increases apparent total tract digestibility coefficients (CATTD) of dry matter, gross energy, crude protein and digestible energy in pigs, but did not result in increases of ADFI or ADG; air classification had little effect on growth performance of weaned pigs [101].

#### Effect of enzyme supplementation

There are few studies that have been conducted to evaluate the effect of NSP-degrading enzymes on digestibility and performance of pigs fed diets supplemented with CM. For instance, Thacker [89] fed barley-based diets containing CM and supplemented with multi-carbohydrase enzymes to growing pigs and found that enzyme had no effect on growth performance and ATTD of nutrients. In a study with weaned pigs, Zijlstra et al. [102] found that carbohydrase supplementation to a wheat and CM based diet improved the ADFI and ADG, but did not improve feed efficiency and ATTD of nutrients. They postulated that carbohydrase enzymes reduced the digesta viscosity thereby increasing the passage rate which led to an increase in ADFI. Zhang et al. [100] reported that when using exogenous multi-enzyme (EME) in piglets 35 to 65 d of age, the values for ATTD of DM, CP, and GE were greater than when piglets were fed diets without EME supplementation. In the performance study it was observed that the ADG, ADFI, and feed efficiency tended to be greater with the increasing levels of supplemented EME, additionally, it was observed that inclusion of EME resulted in increased counts of *Lactobacilli* spp. and *Bacillus subtilis* spp., and reduced the population of *Salmonella* spp. and *Escherichia coli* spp. in the feces. The activities of amylase, lipase, and protease in the small intestine were enhanced with the inclusion of EME in the diets [100]. A greater impact of enzyme supplementation on nutrient utilization of CM has been observed with the use of phytase, for example, in a phosphorus digestibility study conducted by Maison et al. [51] it was reported that supplemental microbial phytase increased ATTD and STTD from 44.99 and 48.82 % to 64.08 and 67.97 % for CM, from 46.77 and 50.36 % to 63.53 and 67.29 % for 00-rapeseed and from 44.83 and 48.60 % to 69.18 and 72.99 % for rapeseed expellers. Adhikari et al. [3] evaluated 2 types of CM and 3 levels of phytase (i.e., 0, 500 and 2,500 U/kg) and observed that as the phytase level incrased, the ATTD of P increased from 39.1 to 69.3, and 78.9 % in treatments containing *B. napus *black meal, and from 46.0 to 71.4 and 78.0 % in treatments containing *B. juncea *yellow meal fed to growing pigs. The STTD of P also increased in similar way as shown in Figure 1.

#### Fermentation

Solid state fermentation (SSF) of CM using *Aspergillus ficuum* has been used to increase the amount of protein and to reduce the amount of phytic acid [62]. Furthermore, Ebune et al. (1995) reported that phosphate and glucose concentration are important factors to consider to maximizing the production of phytases and the reduction of phytic acid content in CM during the SSF process using *A. ficuum.* The use of *Lactobacillus salivarius* in SSF of CM has resulted in decrease in the amount of GSL, crude fiber (CF), and the increase of CP content [4].

Aljuobori et al. [6] selected traditional foods fermented by microorganisms naturally present in food and isolated lactic acid bacteria (LAB); from the isolates obtained it was determined that most of them were *Lactobacillus* and 10 of them were selected to ferment CM, being *Lactobacillus salivarius* the most efficient LAB to reduce the total GSL and CF content of CM which reported reductions from 22.0 to 13.6 % and from 12.0 to 10.1 %, respectively. Such values are slightly lower than those reported by Pal Vig and Walia [70], in research of solid state fermentation in rapeseed meal using *Rhizopus oligosporus,* the study reported a reduction of GSL and CF by 43.1 and 25.5 %, respectively.

### Utilization of canola meal in swine feed

Canola meal can be used as a cost effective protein substitute for other protein sources such as soybean meal in pig diets. Depending on its relative nutritive value and cost, it is economical to replace soybean meal partially or fully with CM. The literature contains enough evidence that CM has been used for more than forty years in swine diets.

#### Starter pig diets

It appears that majority of the studies on CM use in starter pig diets were mainly focused on growth performance. In the past, it was suggested that complete [54] or partial [26] replacement of soybean meal with CM had negative effects on pig performance [11]. It was also documented that increasing inclusion of CM linearly reduced ADG and ADFI in weaned pigs [8]. In a preference trial, weaned pigs were offered a choice between a SBM based control diet and CM at 5-20 % inclusion level, results indicated that pigs preferred to eat the SBM based control diet more than any of the diet containing CM [7]. There was also a significant reduction in the amount of feed consumed when CM inclusion level was increased from 5 to 20 %. The possible reason for the low intake of a diet containing CM by starter pigs may be the influence of GSL breakdown products on thyroid function and the reduced palatability due to the presence of GSL and their break down products [54].

However, recent findings are contrary to the results of past research. For instance, a recent study reported that either solvent-extracted canola meal (SECM) or expeller-pressed canola meal (EPCM) at 150 g/kg inclusion level combined with crude glycerol can partially replace SBM and wheat in weaned pig diets [79]. In another study, Landero et al. [45] fed 0, 50, 100, 150 and 200 g SECM / kg, in replacement for soybean meal to weaned pigs and found that from 0 to 28 days on trial, increasing inclusion of SECM up to 20 %, did not affect body weight gain, feed intake and feed efficiency, although, increasing inclusion of CM reduced linearly the ATTD of energy, DM and CP and quadratically the DE content of the diets. Landero et al. [46] also conducted another experiment to determine the effect of feeding increasing levels of expeller-pressed canola meal (EPCM) up to 200 g/ kg diet to weaned pigs and found no significant differences in growth performance although there were linear reductions in ATTD of DM, energy and CP. In a more recent study, Sanjayan et al. [75] demonstrated that SECM from *B. napus* black and *B. juncea yellow* can be included in the weaned pig diets at up to 25 % without adverse effect on the growth performance (Table 7). In another study, Mejicanos [56] evaluated high levels of inclusion of parent and dehulled *B. napus* and *B. juncea* CM replacing SBM at 15 % level and found increased growth performance when using Fine 2 dehulled CM. There were two possible explanations proposed for the improved performance of weaned pigs at high CM inclusion. Firstly, in the past, diets were formulated mainly based on CP and DE and not on SID AA or NE. Zijlstra and Payne [103] suggested that formulating diets with by-products as alternative feedstuffs would minimize the risk associated with reductions in growth performance if the NE and SID AA systems were used. The second reason is that recent cultivars of CM have comparatively low amounts of GSL compared to old cultivars [45, 56].

**Table 7:** Effect of dietary canola meal inclusion and canola meal type on nursery pig performance^a^

Item	Control	*B. juncea* yellow		*B. napus* black	
	0 %	20 %	25 %	20 %	25 %
ADG, g/d	400	385	390	395	391
ADFI, g/d	617	607	620	622	618
G:F	063	0.64	0.63	0.64	0.63

^a^piglets were fed canola meal containing diets in two phases for 28 days starting from weaning at 21 d of age. There was no effect of inclusion level or canola meal type. (Adapted from [100])

#### Grower - finisher pig diets

Previous studies reported that CM can be used to replace only up to 50 % of the supplemental protein from SBM in grower pigs [54]. However, replacement of 75 % or complete replacement of SBM by CM significantly reduced the growth performance [8]. Sauer et al. [76] indicated that lower DE and lysine contents in CM compared to SBM and the effect of GSL on feed intake and metabolic process might be the possible reasons for the low performance in grower pigs. Thacker [88] suggested that good performance could be achieved in grower pigs, if CM supplies only one half of the supplementary protein in the diet. In a review on CM, Schöne et al. [77] suggested that growing pigs can tolerate a maximum level of 2 μmol/ g of GSL in the diet. But the total GSL content of Canadian CM is around 7.2 μmol/g [67], which implies a maximum level of 33 % CM in growing pig diet.

Studies to determine the digestibility of nutrients of CM has been conducted, for instance, Bell et al. (1998) reported that *B. napus* black and *B. juncea* yellow had similar digestible protein and energy in finisher pigs. An experiment using toasted and non-toasted black and yellow seeded *B. napus* and yellow *B. juncea* in grower pigs suggested that DE and NE content of *B. napus* yellow seeded is higher than that of conventional *B. napus* black and *B. juncea* [60]. National Research Council [65] indicates NE value for SECM from black *B. napus* to be 1890 kcal/kg, meanwhile, Heo et al. [38] indicates that NE for yellow seeded *B. napus* averaged 2102 kcal/kg, while values for yellow *B. juncea* averaged 2340 kcal/kg, The SID of AA of SECM and EPCM in grower pigs has been reported by several studies [50, 75, 79, 96]. In the mentioned studies, EPCM had greater digestible AA compared to SECM, as can be seen in Table 3.

Previous studies also indicated that CM can be included in pig diets without affecting growth performance and carcass characteristics of the finisher pigs. A performance study was conducted in grower pigs with decreasing amount of expeller extracted CM (22.5, 15, 7.5, and 0 %) to validate the performance and carcass characteristics [79]. Increasing the inclusion level of expeller extracted CM did not affect carcass characteristics such as back fat thickness, loin depth, jowl fat and fatty acid profile; however ADG was reduced by 3 g/day per 1 % inclusion of EPCM. Zanotto et al. [99] fed 20 %, 40 %, 60 % and 80 % of CM, in replacement of soybean meal to growing finishing pigs and found quadratic treatment effect on the weight gain. These authors found that substitution level of 40 % soybean meal yields high weight gain and heavier carcass; although it had greater back fat depth. [9, 64]. Busboom et al. [18] found that canola feeding not only increased the proportion of unsaturated fatty acid in adipose tissue and muscle tissue, but it also didn’t affect the carcass characteristics.

#### Sow diets

Spratt and Leeson [84] evaluated the effects of inclusion of raw ground full fat canola on sow milk composition and piglet growth using *B. napus* (Tower) at levels from 5 to 25 % commencing on 109 day of gestation and continuing until 21 days postpartum, the sow performance was not affected by the use of 5 and 10 % canola seed level but at 15 % a decrease in daily weight gain was observed, resulting on the loss of weight on sows from 7-21 days postpartum, but milk was not affected. More recently, King et al. [42] evaluated the effect of diets containing up to 20 % of SECM on sow performance; results indicated that average sow performance and piglet weight was not affected by the different levels of CM in the diets. In another study Clowes et al. [31] evaluated phase-feeding protein to gestating sows over three parities; the study used CM at a rate of up to 8.1 % and didn’t find effect on maternal growth, piglet birth-weight, and litter growth in lactation, wean-to-breeding interval, or subsequent litter size. Quiniou et al. [71] studied the effects of feeding 10 % of low-glucosinolate rapeseed meal (*B. napus*) during gestation and lactation, over three reproductive cycles, on the performance of hyper prolific sows and their litters and found no differences when compared to diets containing no rapeseed meal. In their study sows farrowed 43.6 and 43.8 piglets over three reproductive cycles, respectively. Piglet weight at birth or weaning survival and litter weight gain were not affected by dietary inclusion of canola meal. Plasma thyroxin levels of sows and piglets indicated that thyroid function was not altered by inclusion of canola of less than 2 μmol/g of GSL. The use of diets containing 10 % of CM on gestation and lactation of hyper-prolific sows over three parities did not affected sow longevity and reproductive and litter performance.

### Practical application of canola meal in swine diets

The energy system used to express requirements for pigs according to NRC 1971 was total digestible nutrients, then metabolizable energy; currently National Research Council [65] expresses AA and nitrogen requirements as standardized ileal digestible and apparent ileal digestible basis, but also they are expressed on total basis, which apply to corn-SBM based diets. In the same way, phosphorus requirements are listed on a STTD, ATTD and total basis. Net energy is also used as the most accurate mean to predict the pigs’ response to energy intake. It is assumed that if the diets are balanced according to SID of AA and net energy, similar performance will be achieved regardless of feedstuff used in the formulation, in that regard, recent research shows that CM can be included in pre-starter and starter diets at levels of 15, 20 and 25 % without affecting pig performance ([45, 56, 75]; Table 7).

Mejicanos [56] evaluated the effect on performance of pigs from 1 to 28 days after weaning when fed diets containing three levels of dehulled CM from *B. napus* black and *B. juncea* compared to control corn-soybean diets. Results show that the type of diet had no effect on ADFI, indicating that pigs readily consumed phase I and Phase II diets containing 15 % of *B. napus* CM and canola quality *B. juncea* meal. Diets containing *B. napus* black increased ADG values compared to *B. juncea* and the corn/SBM based control diet. Overall in the experiment, feed efficiency was increased when pigs were fed diets containing *B. napus* CM*,* observing values of 0.67 compared to 0.62 and 0.58 when pigs were fed diets containing B*. juncea* and corn/SBM respectively. All diets containing *B. napus* black outperformed diets containing *B. juncea* or corn-soybean meal for final BW.

Sanjayan et al. [75] evaluated 20 and 25 % inclusion levels of CM from *B. napus* and *B. juncea* with and without multi-carbohydrase supplementation and found that regardless of variety and inclusion level, there were no significant differences among treatments for ADG, ADFI and G:F ratio for 4 weeks after weaning.

## Conclusion

The current review offers a description of how canola meal has evolved in recent years, the differences between current canola seeds especially with regards to its nutritive value, particularly protein and fiber content which offers improved profiles for animal nutrition in the case of yellow seeded cultivars (i.e. yellow seeded *B. napus* and yellow *B. juncea*).

CM offers an alternative in swine diet as it is a cost effective protein source. This literature review provides information about the nutritive value of CM and recent techniques (i.e., development of new canola cultivars, dehulling of CM and supplementation of feed enzymes and fermentation) which have been used to improve the nutritive value of CM and overcome the limitations encountered by the swine industry and its use as feedstuff. Determination of SID of AA of new cultivars of canola is very important in order to formulate the diet efficiently thereby helping to achieve predictable growth performance in pigs. Furthermore, enzyme supplementation to cereal based diets has yielded inconsistent results.
